# HoNOSCA-D As a Measure of the Severity of Diagnosed Mental Disorders in Children and Adolescents—Psychometric Properties of the German Translation

**DOI:** 10.3389/fpsyt.2017.00186

**Published:** 2017-09-28

**Authors:** Agnes von Wyl, Stephan Toggweiler, Ruedi Zollinger

**Affiliations:** ^1^School of Applied Psychology, Zurich University of Applied Sciences, Zurich, Switzerland; ^2^Medical Center Maienfeld, Maienfeld, Switzerland

**Keywords:** HoNOSCA-D, German-language version, mental disorder, ICD-10, children and adolescents, psychometrics, validity

## Abstract

The Health of the Nation Outcome Scales for Children and Adolescents (HoNOSCA), in use worldwide, is a 13-item measure assessing the biopsychosocial severity of mental health problems in children and adolescents. This article introduces the authorized German-language version of HoNOSCA, the HoNOSCA-D, and examines and discusses its psychometric properties based on a clinical sample of 1,533 children and adolescents aged 4;0 to 17;11 years. For the HoNOSCA-D total score (severity of mental health problems), internal consistency (Cronbach’s alpha) was 0.63. The discriminative power of the items ranged from 0.07 to 0.44; the average interitem correlation was 0.11. Due to this stochastic independence, calculation of a total severity index is acceptable. Using factor analysis, the principal axis factoring and varimax rotation resulted in a four-factor structure, which with a Kaiser–Meyer–Olkin measure of sampling adequacy of 0.684 explained 30.62% of total variance. The convergent correlations with the German-language parent report version of the Strengths and Difficulties Questionnaire were as expected and showed a medium effect size. Gender and age differences in the HoNOSCA-D total score were small. Regarding the 13 items gender and age differences were negligible to medium. The highest severity was found for schizophrenia and psychotic disorders, followed by affective disorders and social behavior disorders. Overall, validity of HoNOSCA-D was clearly supported.

## Introduction

### Reason for the Study and Theoretical Background

Child and adolescent psychiatric services are increasingly confronted with demands for evidence of the effectiveness of their interventions in terms of outcome. For German-speaking countries, Mattejat and Remschmidt [e.g., Ref. (1)] provide various concepts for systematic collection of outcome data. For English-speaking countries, Bickman et al. (2) looked at various approaches and developed recommendations for a modular system of outcome measurement. Here they found the *Health of the Nation Outcome Scales for Children and Adolescents* (HoNOSCA), developed by Gowers et al. (3) in England, particularly important. HoNOSCA is based on the *Health of the Nation Outcome Scales* (HoNOS) (4, 5), which were developed in the United Kingdom in the early 1990s as a clinician-rated assessment instrument for routine use in adult mental health services. A major aim was the construction of a practicable instrument for differentiated assessment of the severity of mental illness that can be used as a routine clinical outcome measure.

Based on a review of the literature by Hunter et al. (6), HoNOSCA was developed taking account of specific needs in child and adolescent mental health, which resulted in an increase in the number of items (scales) from 12 to 13. Each of these biopsychosocial indicators flags mental health problems and can determine whether the young person will be referred to psychiatric care. Like HoNOS, HoNOSCA is a clinician-rated instrument for assessing the severity of mental illness. Hunter et al. (6) emphasized that this kind of graduated rating of severity complements a psychiatric diagnosis and is of central importance with regard to outcome and measurement of the course of illness. The 13 items, or scales, assess different areas of symptoms and functioning: *Behavior, Impairments, Symptoms*, and *Social*. Two additional items concern parents’ lack of understanding or lack of information about difficulties and about therapy, services, and other help and arrangements offered in connection with the patient’s mental health problems. Ratings on these two items are not a part of the total score calculated for items 1–13.

### HoNOSCA Goodness Criteria

Regarding interrater reliability, previous studies found that the total score for the 13 items shows good-to-very good agreement, with intraclass correlations from 0.63 to 0.98 (3) [see also Ref. (7)]. Noteworthy is a study by Hanssen-Bauer et al. (8), which reported an intraclass correlation of raters in five countries of 0.84. For retest reliability, Harnett et al. (9) found, with a time interval of 2–3 weeks, a correlation between the total scores of *r* = 0.80. But there are also less favorable findings: Brann et al. (10) found a very low intraclass correlation (ICC = 0.06) for one item and only moderate intraclass correlations (ICC = 0.43) for three items. This discrepancy underlines the importance of careful rater training.

To assess the construct validity of HoNOSCA various methods have been used. Comparing HoNOSCA to other structured clinician ratings, Bilenberg (11) found associations between the HoNOSCA total score and the *Global Assessment of Psychosocial Disability* (GPAD) (12) of *r* = 0.62 (*p* < 0.001, *N* = 173). Yates et al. (13) reported an association of *r* = −0.64 (*p* < 0.01, *N* = 230) with the *Children*’*s Global Assessment Scale* (CGAS) (14) and a correlation of *r* = 0.62 (*p* < 0.01, *N* = 248) with the *Paddington Complexity Scale* (PCS) (13). For the PCS, Harnett et al. (9) found a somewhat lower correlation of *r* = 0.46 (*p* < 0.01, *N* = 51) and *r* = −0.47 (*p* < 0.01, *N* = 51) with HoNOSCA rates of change over a 3-month period. Looking at parent ratings, Yates et al. (13) found a correlation of *r* = 0.44 (*p* < 0.01, *N* = 37) with the *Behavior Checklist* (15, 16) and a correlation of *r* = 0.40 (*p* < 0.001, *N* = 208) with the *Strengths and Difficulties Questionnaire* (SDQ) (17), which we also used in this study.

Factor analysis of the four areas of functioning suggested by Gowers et al. (3, 9) yielded less unambiguous results, but no convincing evidence has since been found for these results.

Regarding criterion-related validity of HoNOSCA, Gowers et al. (3) tested whether HoNOSCA could differentiate between different patient groups. They found evidence of this insofar as they found that for clients in inpatient psychiatric treatment, who made up approximately 12% of the sample of *N* = 1,143, the HoNOSCA total score was *M* = 15.51 (SD = 7.19), but for clients in outpatient treatment, the total score was *M* = 11.18 (SD = 5.30). This difference was statistically significant [*t*(1 141) = −8.03, *p* < 0.001], and the effect size was nearly medium. As to age differences in HoNOSCA total scores, in a child and adolescent mental health service sample from the Melbourne area, Brann et al. (10) found significant differences among three age groups (*n*_<5 years_ = 34, *n*_5–12 years_ = 142, *n*_>12 years_ = 129) with a medium effect size (η^2^ = 0.095). *Post hoc*, this significance with a medium effect size (*d* = 0.70) was ascribable to the difference between the 13- to 20-year age range (*M* = 15.21, SD = 6.66, *n* = 113) and the 5- to 12-year age range (*M* = 11.11, SD = 5.15, *n* = 125). In contrast, Harnett et al. (9) found no age differences.

At the level of individual HoNOSCA items, Kisely et al. (18) found that children younger than age 12 years had significantly lower scores than older children, with medium effect size, on the items *non-accidental self injury* and *alcohol, substance/solvent misuse*.

For children and adolescents aged 0–20 years (*N* = 173), Bilenberg (11) found that in patients with comorbidity (*n*_1_ = 77), both the HoNOSCA total score baseline and the follow-up measurement 3 months later or at the end of therapy were significantly higher than in comparable patients without comorbidity (*n*_2_ = 96).

In studies with longitudinal designs, Kisely et al. (18) found significant changes over time in HoNOSCA total scores for both outpatients and inpatients. For inpatients (*n*_1_ = 30) the effect size was very large (*d* = 1.34; with an average measurement interval of 24.3 days); for outpatients (*n*_2_ = 123) the effect size was small (*d* = 0.32; with an average measurement interval of 45.5 days). Harnett et al. (9) reported similar results. Quantified in raw scores, Gowers et al. (3) found a 38% reduction in the total score; this was after an average treatment duration of 3 months and in a sample of several hundred persons. Where clinicians reported definite improvement of the mental health problem, an average change in the HoNOSCA total score of *M*_d_ = 7.70 (SD_d_ = 5.40) was found. Where clinicians reported no change, the average change was only *M*_d_ = 1.60 (SD_d_ = 4.99), and where clinicians diagnosed a worsening of the mental health problem, the average change in the HoNOSCA total score was found to be *M*_d_ = −1.00 (SD_d_ = 4.53).

Bilenberg (11) reported on the usefulness of HoNOSCA in the context of a Danish field trial; about 80% of the clinicians surveyed found HoNoSCA to be clinically feasible and easy and fast to use. Gowers et al. (3) reported a mean time to complete the scale of 8.5 min.

### Aims of This Study

The aim of this study was to report initial results on item characteristics and validity of the German-language version of HoNOSCA, the HoNOSCA-D, based on the following analyses:

– Item and scale analyses, homogeneity, internal consistency.
– Correlations with the parent report version of the SDQ (17, 19).
– Exploratory factor analysis (principal axis factoring and varimax rotation).
– Psychiatric diagnoses and HoNOSCA-D scores.
– Group differences for gender and age.

The first three analyses above concern the internal consistency and construct validity of HoNOSCA-D, and the last two pertain to criterion-related validity.

## Materials and Methods

### German Translation of HoNOSCA

Health of the Nation Outcome Scales for Children and Adolescents, consisting in a score sheet and user guide, was translated by the first author of this article using the World Health Organization translation guidelines. HoNOSCA-D was then practice-tested at two offices of the child and adolescent psychiatric services of the Canton of St. Gallen (KJPD SG) in Switzerland for a period of 6 months. Clinicians’ ideas and suggestions for changes were recorded and then reviewed by a group of clinical experts, and some of them were incorporated in a revised version. The revised version of HoNOSCA-D was then back-translated by a native speaker of English and sent to the authors of HoNOSCA (Simon Gowers) for authorization.

### Setting and Design

The study was approved by the *Eidgenössische Expertenkommission für das Berufsgeheimnis in der medizinischen Forschung* (Swiss federal expert committee for professional confidentiality in medical research), which was also responsible for ethical approval. The data were collected from May 2010 and April 2012 in the context of a naturalistic field study at the KJPD SG. The KJPD SG is responsible for basic psychiatric services in a region with a population of approximately 500,000 persons, about one-fifth of whom are children and adolescents. The scales were part of a systematic outcome measurement and were used for both outpatients and inpatients aged 4;0 to 17;11 years. For all new patients, the case manager (psychiatrist, psychologist, or social worker) had to fill out HoNOSCA-D and decide on the diagnosis within the first two in depth consultations. The ICD-10 diagnosis was given then in agreement with the senior physician. The parents of all new patients received the SDQ questionnaire, which they usually filled out on-site and immediately before the first meeting. This interrogation is part of the usual intake procedure and is related to the diagnostic process and quality assurance. Parents usually were informed that if they don’t want to fill out an anonymized SDQ and therefore contribute to the quality assurance one actively has to pull out from the survey.

### Measurement Instruments

#### HoNOSCA-D

The following items make up HoNOSCA-D: 1. *Störendes, asoziales oder aggressives Verhalten* (disruptive, antisocial, or aggressive behavior), 2. *Überaktivität, Aufmerksamkeit und Konzentration* (overactivity, attention, or concentration), 3. *Absichtliche Selbstverletzungen* (non-accidental self injury), 4. *Alkohol, Suchtmittel oder Lösungsmittelmissbrauch* (alcohol, substance/solvent misuse), 5. *Schulische oder sprachliche Fähigkeiten* (scholastic or language skills), 6. *Körperliche Erkrankung oder Behinderung* (physical illness or disability), 7. *Halluzinationen und Wahnvorstellungen* (hallucinations, delusions, or abnormal perceptions), 8. *Nicht organisch bedingte somatische Symptome* (non-organic somatic symptoms), 9. *Emotionale und zugehörige Symptome* (emotional and related symptoms), 10. *Beziehungen zu Gleichaltrigen* (peer relationships), 11. *Selbstpflege und Unabhängigkeit* (self-care and independence), 12. *Familienleben und familiale Beziehungen* (family life and relationships), 13. *Geringe Beteiligung an der Schule* (poor school attendance).

The items cover four different areas of symptoms and functioning: *Behavior* (items 1–4), *Impairments* (items 5–6), *Symptoms* (items 7–9), and *Social* (items 10–13) and are rated by the clinician on a 5-point scale of severity (0 = *no problem*, 1 = *minor problem requiring no action*, 2 = *mild problem but definitely present*, 3 = *moderately severe problem*, and 4 = *severe-to-very severe problem*). For each item the rating format is complemented by item-specific criteria for including and not including certain problems and behaviors in the rating. For each item the most severe known problem has to be rated, and the rater may use all available information (such as information from the referring institution, from teachers, etc.) for forming a judgment. If the rater does not know the severity of a particular item, or if the item is not applicable, the rater rates the item “9,” and it is not used further. The HoNOSCA-D total score represents the summed severity of individual items 1–13. For the entire procedure (conducting, rating, and interpretation), the clinicians in this study had access to a comprehensive manual, had 1 day of training and every 3 months a refresher of 1–2 h with the opportunity to ask questions. Each of this units was held by the first author of this article.

#### German Version of the SDQ

The German Strengths and Difficulties Questionnaire (SDQ-Deu) for parents (17, 19–21), was used as this measurement is widely used to assess informant ratings on psychological problems. It contains the scales *emotional symptoms* [α = 0.66; see Ref. (22)], *hyperactivity/inattention* (α = 0.76), *peer relationship problems* (α = 0.58), *conduct problems* (α = 0.60), and *prosocial behavior* (α = 0.68). Each scale has five items rated on a 3-point scale (0 = *not true*, 1 = *somewhat true*, 2 = *certainly true*). The total difficulties score (α = 0.82) is the sum of all the scales except the *prosocial behavior* scale.

### Data Analyses

To evaluate the item and scale characteristics of HoNOSCA-D, after analysis of missing values, descriptive characteristic values were calculated and item and scale analyses carried out. The average interitem correlation (homogeneity) was calculated using Fisher’s *z* transformation. For construct validity, we computed Pearson correlations with the parent SDQ. We also carried out a principal axis factor analysis with varimax rotation. For criterion-related validity, we used a 2 × 3 ANOVA. To minimize statistical inference type 1 errors due to alpha inflation, we used Bonferroni correction of the level of significance. As a measure of effect sizes (23), for the correlations, we used their *r* measure (small: *r* = 0.1; medium: *r* = 0.3; large: *r* = 0.5) and for the ANOVA η^2^ (small: η^2^ = 0.01; medium: η ^2^ = 0.06; large: η^2^ = 0.14).

## Results

### Sample

Table 1 shows the composition of the sample.

**Table 1:** HoNOSCA-D sample (*N* = 1,553).

	Frequency (%)
**Gender**	
Female	664 (42.8)
Male	889 (57.2)
**Age**	
*M* (SD)	12.06 (3.70)
Range	4.01–17.98
Skewness/kurtosis	−0.29/−0.98
**Ethnical background**	
Swiss^a^	1,208 (77.8)
Rest of West-/North Europe	101 (6.5)
East- and South East Europe	57 (3.7)
South Europe	123 (7.9)
Asia, Africa, and South America	35 (2.3)
Not specified	29 (1.9)
**Education father**	
None	8 (0.5)
Compulsory education	31 (2.0)
Diploma of vocational and educational training	309 (19.9)
Baccalaureate	4 (0.3)
(Advanced) federal diploma of higher education	57 (3.7)
University	46 (3.0)
Unknown	192 (12.4)
**Diagnosis**	
Schizophrenia/delusional disorder F2	13 (0.8)
Affective disorders F3	75 (4.8)
Adjustment disorders F43	306 (19.7)
Neurotic disorders F4 without F43	69 (4.4)
Eating disorders F50	15 (1.0)
Pervasive developmental disorders F84	16 (1.0)
Hyperkinetic disorders F90	197 (12.7)
Conduct disorders F91	44 (2.8)
Mixed disorders of conduct and emotions F92	44 (2.8)
Emotional disorders with onset specific to childhood F93	298 (19.2)
Other conduct and emotional disorders F98	75 (4.8)
Other diagnoses	76 (4.9)
Main diagnosis on axis III–VI	96 (6.2)
Unknown	229 (14.7)

*^a^At least one parent Swiss*.

The HoNOSCA-D total sample of *N* = 1,553 contains, as a paired sample with *n* = 1,408, the sample of the parent SDQ. The difference is missing data.

### Item and Scale Analyses, Homogeneity

Concerning difficulty index *P* (Table 2), there was a wide range, starting with items with low difficulty index values: *hallucinations, delusions, or abnormal perceptions*; *alcohol, substance/solvent misuse*; and *non-accidental self injury*. It is noteworthy that for every item the entire range of five points was used; there were no missing values.

**Table 2:** Item and scale analyses, exploratory factor analysis (principal axis factor and varimax rotation) and intercorrelations (*N* = 1,553).

		Item and scale analyses			Factor loadings^a^				Intercorrelations^b^											
	HoNOSCA-D Item	*M* (SD)	*P*	*r*_it_	1	2	3	4	1	2	3	4	5	6	7	8	9	10	11	12
1	Problems with disruptive, antisocial, or aggressive behavior	1.17 (1.16)	0.29	0.37	0.32	0.61														
2	Problems with overactivity, attention, or concentration	1.44 (1.24)	0.36	0.28	0.51				0.33											
3	Non-accidential self injury	0.29 (0.74)	0.07	0.15																
4	Problems with alcohol, substance/solvent misuse	0.22 (0.64)	0.06	0.13				0.77	0.15		0.21									
5	Problem with scholastic or language skills	1.25 (1.23)	0.31	0.35	0.67				0.21	0.39										
6	Physical illness or disability problems	0.32 (0.85)	0.08	0.16									0.13							
7	Problems associated with hallucinations, delusions, or abnormal perceptions	0.09 (0.44)	0.02	0.07							0.13									
8	Problems with non-organic somatic symptoms	0.78 (1.09)	0.20	0.08			0.33		−0.10											
9	Problems with emotional and related symptoms	2.21 (1.09)	0.55	0.32		0.38	0.69				0.21			0.10		0.21				
10	Problems with peer relationships	1.39 (1.20)	0.35	0.39	0.40				0.25	0.17			0.25	0.10			0.29			
11	Problems with self care and independence	0.63 (0.97)	0.16	0.44	0.53				0.22	0.24			0.34	0.19			0.17	0.31		
12	Problems with family life and relationships	1.91 (1.20)	0.48	0.29		0.48			0.30		0.11	0.11					0.30	0.13	0.22	
13	Poor school attendance	0.69 (1.17)	0.17	0.36	0.34				0.23	0.12		0.18	0.25			0.10	0.17	0.22	0.24	0.17
	HoNOSCA-D total score	12.41 (5.76)	0.24																	

*^a^Loadings ≥ |0.30|. Kaiser–Meyer–Olkin (KMO) measure of sampling adequacy = 0.684, Bartlett-test chi-square = 2,156.79, *df* = 78, *p* < 0.001, explained overall variance = 30.62%*.

*^b^After Bonferroni correction (alpha/78) only at *p* < 0.00064 significant correlations are shown*.

The HoNOSCA-D total score was skewed to the left (skewness = 0.82, kurtosis = 0.84); difficulty index value was low, at 0.24. Discrimination of 7 of the 13 items was below 0.30. Internal consistency (Cronbach’s α) of the total score was 0.63.

In all, only 6 of 78 intercorrelations had at least a medium effect size of more than 0.3; the other effect sizes were small. After Fischer’s *z* transformation, the homogeneity of the scale was 0.11.

### Correlations with the Parent SDQ

We correlated HoNOSCA-D with the parent SDQ (17, 19–21) (Table 3); the significance values for the 84 individual comparison were Bonferroni adjusted.

**Table 3:** Pearson correlations with the parent report version of the SDQ (*n* = 1,408).

	SDQ parent report					
HoNOSCA-D	Emotional problems	Conduct problems	Hyperactivity	Peer relationship problems	Prosocial behavior	Total difficulties score
1. Disruptive, antisocial, or aggressive behavior		0.45	0.32	0.18	−0.28	0.32
2. Overactivity, attention, or concentration		0.19	0.42	0.15		0.27
3. Non-accidental self injury	0.15					0.10
4. Alcohol, substance/solvent misuse		0.10			−0.13	
5. Scholastic or language skills			0.18	0.13		0.14
6. Physical illness or disability						
7. Hallucinations, delusions, or abnormal perceptions						
8. Non-organic somatic symptoms	0.26	−0.10				
9. Emotional and related symptoms	0.31		−0.15			
10. Peer relationships	0.14	0.09		0.49	−0.15	0.28
11. Self-care and independence			0.10	0.15	−0.13	0.13
12. Family relationships		0.16			−0.12	0.12
13. Poor school attendance		0.10				0.12
Total score	0.16	0.22	0.19	0.28	−0.18	0.32

*The significance levels of the correlations are Bonferroni corrected (alpha/84); all reported values are at *p* < 0.00008 highly significant*.

Of the correlations with at least medium effect size, noteworthy is the HoNOSCA-D item *peer relationships*, which was correlated with SDQ *peer relationship problems*, followed by *disruptive, antisocial or aggressive behavior*, which was correlated with SDQ *conduct problems* and *hyperactivity* and the SDQ total difficulties score. The HoNOSCA-D item *overactivity, attention or concentration* was correlated with SDQ *hyperactivity*, and *emotional and related symptoms* was correlated with SDQ *emotional problems*. Regarding assessment of the HoNOSCA-D total score, it was found to be moderately correlated with the SDQ total difficulties score.

### Exploratory Factor Analysis (Principal Axis Factor)

Principal axis factoring and varimax rotation (Table 2)—with a mediocre Kaiser–Meyer–Olkin measure of sampling adequacy of 0.684, an eigenvalue of 1, and explained total variance of 30.62%—produced a four-factor solution.

### HoNOSCA-D Scores and Psychiatric Diagnoses

The psychiatric diagnoses were combined in diagnostic groups, which do not consistently follow the single-digit mental disorders groups of the ICD-10, however (Table 5). The aim was to form meaningful evaluation categories in view of the HoNOSCA-D items.

Table 4 shows that for each disorder there was at least one HoNOSCA-D correspondence with a severity rating of at least 2 (2 = *mild problem but definitely present*). *Emotional and related symptoms* played a leading role here, whereby they were particularly severe for ICD-10 F50 *Eating disorders* and F3 *Affective disorders*. In second place were problems in *family life and relationships*. On the basis of the HoNOSCA-D total score, ICD-10 *F2 Schizophrenia, schizotypal and delusional disorders*, followed by F92 *Mixed disorders of conduct and emotions* and F3 *Affective disorders*, were the most severe disorders.

**Table 4:** HoNOSCA-D total score and psychiatric diagnosis (*n* = 1,093).

	*n*		Disruptive, antisocial, or aggressive behavior	Overactivity, attention, or concentration	Non-accidental self-injury	Alcohol, substance/solvent misuse	Scholastic or language skills	Physical illness or disability	Hallucinations, delusions, or abnormal perceptions	Non-organic somatic symptoms	Emotional and related symptoms	Peer relationships	Self-care and independence	Family relationships	Poor school attendance	Total score
Schizophrenia/delusional disorder F2	13	*M*									2.31			2.62		17.77
		SD									1.18			1.26		7.52
Affective disorders F3	75	*M*									3.03			2.07		15.01
		SD									0.81			1.16		5.71
Neurotic disorders F4 without F43	69	*M*									2.67					12.70
		SD									1.02					5.85
Adjustment disorders F43	306	*M*									2.25			2.14		11.25
		SD									0.94			1.20		5.50
Eating disorders F50	15	*M*									3.13					10.93
		SD									0.74					4.50
Disorders of psychological development F8 without F84	16	*M*					2.19									8.69
		*SD*					1.05									4.32
Pervasive developmental disorders F84	16	*M*									2.44	2.56				13.19
		SD									0.89	1.41				7.88
Hyperkinetic disorders F90	197	*M*		2.69												14.04
		SD		0.88												5.96
Conduct disorders F91	44	*M*	2.16											2.32		13.11
		SD	1.14											1.05		6.12
Mixed disorders of conduct and emotions F92	44	*M*	2.39								2.55			2.77		17.16
		SD	0.87								0.98			0.80		5.13
Emotional disorders with onset specific to childhood F93	298	*M*									2.26					11.58
		SD									1.06					4.89

*Values ≥2 are shown (*mild problem but definitely present*). *n*_(other diagnoses)_ = 460*.

### Group Differences in Gender and Age

As mentioned in the note, the significance level in Table 5 is Bonferroni adjusted. Regarding age differences, the item score increased, if significant, steadily across the three age groups, whereby adolescents had a significantly highest score, unless *overactivity, attention, or concentration*, which had a significant lower score than the other two groups. The effect sizes of these main effects are negligible to small, with the exception of *alcohol, substance/solvent misuse* which shows a medium effect size. Concerning gender differences there show up only two significant main effects which are *disruptive, antisocial or aggressive behavior* [*F*(1, 1,547) = 24.51***, part. η^2^ = 0.02] and *overactivity, attention or concentration* [*F*(1, 1,547) = 29.15***, part. η^2^ = 0.02]. In both cases, boys reach significant higher values than girls. The HoNOSCA-D total score is equal between girls and boys [*F*(1, 1,547) = 8.17]. Supplementary there appears one significant interaction within *non-accidental self injury* [*F*(2, 1,547) = 10.08***, part. η^2^ = 0.01], though the effect is small.

**Table 5:** HoNOSCA-D age and gender differences.

	4;0–5;11 years (*n*_1_ = 104)	6;0–11;11 years (*n*_2_ = 625)	12;0–17;11 years (*n*_3_ = 824)				Female (*n*_f_ = 664)	Male (*n*_m_ = 889)			Age by gender interaction
								
HoNOSCA-D item	*M*_1_ (SD_1_)	*M*_2_ (SD_2_)	*M*_3_ (SD_3_)	*F*-Value	Part. η^2^	Scheffé (*p* < 0.05)	*M*_f_ (SD_f_)	*M*_m_ (SD_m_)	*F*-Value	Part. η^2^	Part. η^2^
1. Disruptive, antisocial, or aggressive behavior	1.23 (1.18)	1.19 (1.11)	1.14 (1.20)	0.39	n.s.		0.89 (1.07)	1.37 (1.19)	24.51	0.02***	n.s.
2. Overactivity, attention, or concentration	1.39 (1.38)	1.65 (1.20)	1.28 (1.22)	7.30	0.01*	2 > 3	1.10 (1.15)	1.69 (1.24)	29.15	0.02***	n.s.
3. Non-accidental self injury	0.10 (0.45)	0.11 (0.42)	0.45 (0.90)	40.75	0.05***	1 = 2 < 3	0.42 (0.89)	0.19 (0.57)	4.51	n.s.	0.01***
4. Alcohol, substance/solvent misuse	0.04 (0.31)	0.00 (0.07)	0.40 (0.83)	74.76	0.09***	1 = 2 < 3	0.22 (0.60)	0.21 (0.66)	0.10	n.s.	n.s.
5. Scholastic or language skills	1.05 (1.19)	1.30 (1.22)	1.24 (1.24)	1.35	n.s.		1.05 (1.20)	1.40 (1.24)	8.06	n.s.	n.s.
6. Physical illness or disability	0.15 (0.62)	0.29 (0.79)	0.37 (0.90)	3.98	n.s.		0.34 (0.85)	0.31 (0.84)	0.03	n.s.	n.s.
7. Hallucinations, delusions, or abnormal perceptions	0.02 (0.20)	0.04 (0.26)	0.14 (0.55)	10.77	0.01***	1 = 2 < 3	0.12 (0.52)	0.07 (0.36)	0.97	n.s.	n.s.
8. Non-organic somatic symptoms	0.67 (1.05)	0.79 (1.10)	0.79 (1.09)	1.15	n.s.		0.91 (1.14)	0.69 (1.05)	3.16	n.s.	n.s.
9. Emotional and related symptoms	1.85 (1.14)	2.04 (1.07)	2.39 (1.07)	19.12	0.02***	1 = 2 < 3	2.42 (1.02)	2.06 (1.12)	4.26	n.s.	n.s.
10. Peer relationships	1.37 (1.29)	1.41 (1.14)	1.38 (1.24)	0.08	n.s.		1.32 (1.21)	1.45 (1.20)	2.29	n.s.	n.s.
11. Self-care and independence	0.61 (0.95)	0.60 (0.92)	0.67 (1.00)	1.18	n.s.		0.56 (0.92)	0.69 (0.99)	3.49	n.s.	n.s.
12. Family relationships	1.70 (1.21)	1.79 (1.22)	2.03 (1.17)	7.70	0.01**	1 = 2 < 3	1.97 (1.18)	1.87 (1.21)	0.02	n.s.	n.s.
13. Poor school attendance	0.31 (0.86)	0.46 (0.89)	0.91 (1.33)	36.64	0.05***	1 = 2 < 3	0.60 (1.10)	0.76 (1.21)	7.89	n.s.	n.s.
Total score	10.48 (5.65)	11.67 (5.33)	13.21 (5.96)	22.35	0.03***	1 = 2 < 3	11.92 (5.46)	12.77 (5.96)	8.17	n.s.	n.s.

Bonferroni corrected level of significance (alpha/14): *p < 0.00385 significant, **p < 0.00077 very significant, ***p < 0.00008 highly significant. *η*^2^: effect size (small: *η*^2^ = 0.01; medium: *η*^2^ = 0.06; large: *η*^2^ = 0.14).

## Discussion

### Item and Scale Analyses

The item and scale analyses (see Table 2) showed that only 6 of 13 items had high enough discrimination for *a priori* justification of scale construction. The interitem correlations and homogeneity were also very low. This resulted in a relatively low internal consistency of 0.63 (Cronbach’s α). Harnett et al. (9) reported an even lower internal consistency of α = 0.45. This situation is actually not sufficient to ascribe satisfactory scale characteristics to HoNOSCA-D for scale construction in the classical sense. However, the finding that the interitem correlations and the homogeneity are so low allows the construction of an index, since a high share of the items are stochastically independent. The result of this is a HoNOSCA total score in which the contributions of the individual items come into effect independently of one another. Based on 13 dimensions, various clinical features in specific domains of functioning are described. As mentioned above, Harnett et al. (9) found it diagnostically useful to capture these various psychosocial and psychiatric features in the items. Gowers et al. (3) also pointed to the low interitem correlations and thus to the independence of the main scales (*r* = 0.13, range = 0.41–0.01). These findings are in line with Hunter et al. (6), who emphasized that HoNOSCA is an instrument that *complements* psychiatric diagnosis by means of an index of severity, meaning the extent of a disorder.

### Validity

#### Correlations with the Parent SDQ

As mentioned above in the introduction, there are high correlations between HoNOSCA and other outcome measures, such as GPAD (12), CGAS (14), and PCS (13). The HoNOSCA total score—that is, the complexity of the mental health problem—was also found to be correlated with therapeutic progress as assessed by therapists (9). For parent ratings, using the parent SDQ Yates et al. (13) found a medium correlation effect of *r* = 0.40. This value is only negligibly higher than the *r* = 0.32 (Table 3) value reported in this study; both correlations are in the range of medium effect size. Also the correlative analysis of the 13 HoNOSCA-D items with the parent SDQ dimensions revealed various associations that confirm that HoNOSCA-D has intact convergent validity, insofar as four dimensions of the parent SDQ including the total difficulties score were moderately correlated with items with related content on HoNOSCA-D. Only the SDQ dimension *prosocial behavior*—which, however, is a strength and is not included in the SDQ total difficulties score—had a significant negative correlation with the HoNOSCA-D items. This means overall that between the parent SDQ and HoNOSCA-D there is expected but not very marked correspondence in the areas to which they apply: The HoNOSCA-D provides a perspective on 13 broad problem indicators, whereas the parent SDQ is meant for analytical distinguishing between 4 and 5 latent dimensions.

#### Factor Analysis

Although we found a four-factor solution, our factor analysis did not confirm the areas of functioning postulated *a priori* by Gowers et al. (3) (Table 2); however, Gowers et al. themselves were not able to confirm that structure through factor analysis.

#### Psychiatric Diagnoses

Next, we evaluated the average HoNOSCA-D scores for some psychiatric diagnoses (11, 24) (Table 4). It is not surprising that ICD-10 F2 *Schizophrenia, schizotypal and delusional disorders* and F3 *Affective disorders*, also together with F92 *Mixed disorders of conduct and emotions*, are the most severe biopsychosocial disorders. Almost always, problems are found in HoNOSCA-D *emotional and related symptoms* but also often in *family life and relationships*. The fact that there are associations between HoNOSCA-D *overactivity, attention or concentration* and ICD-10 F90 *Hyperkinetic disorders* and between HoNOSCA-D *peer relationships* and F84 *Pervasive developmental disorders* is plausible. Equally plausible associations are found between HoNOSCA-D *disruptive, antisocial or aggressive behavior* and F92 *Mixed disorders of conduct and emotions* and F91 *Conduct disorders* and between HoNOSCA-D *scholastic or language skills* and F8 *Disorders of psychological development* without F84 *Pervasive developmental disorders*. There was insofar an inconspicuous picture concerning the average HoNOSCA-D scores across the different disorders. In the Danish field trial, Bilenberg (11) found a substantial correlation between clinical severity (captured through diagnosis) and mean HoNOSCA scores. In this study, we also found that correlation, which confirms the validity of the rating.

#### Gender and Age Differences

This study found small gender differences. Similar to Hanssen-Bauer et al. (8), boys had significantly higher HoNOSCA-D scores as girls in the two externalizing scales *disruptive, antisocial, or aggressive behavior* and *overactivity, attention, or concentration*. However, unlike Bauer et al. (8), in our sample the difference in *scholastic or language skills* was not significant. Although not significant, there was a tendency for girls to show higher scores on internalizing scales like *non-organic somatic symptoms* and *emotional and related symptoms*. Again, this is similar to Hanssen-Bauer et al. (8), who found higher scores for *emotional symptoms* and *non-accidental self-injury* for girls. In our sample, girls in the oldest age group had significant higher scores for *self-injury* than boys.

A look at HoNOSCA-D scores across the three age groups (Table 5) reveals that adolescents have a significant and noteworthy higher score than the two younger age groups on six scales: *self-injury, drug or alcohol misuse, abnormal thoughts or perceptions, emotional symptoms, family problems* and *poor school attendance*. The highest scores in adolescents for the scales *self-injury, drug or alcohol misuse, abnormal thoughts or perceptions, family problems*, and *poor school attendance* are understandable. Hanssen-Bauer et al. (8) found three of them as well: *self-injury, drug or alcohol misuse*, and *poor school attendance*. However, the highest scores for adolescents are less obvious for *emotional symptoms*. The middle age group (6;0–11;11) showed more difficulties with *overactivity and concentration* than the adolescents.

If we take into account the factors that can be responsible for the differences found for gender and age, it becomes difficult to use these variables as correlates for criterion-related validity. Harnett et al. (9) named various aspects that can cause the discrepancies, such as: different access to alcohol and drugs, practices in admittance to inpatient or outpatient treatment settings, sociodemographic characteristics of the population receiving mental health services, the available age-, gender-, and problem-specific mental health services, at what time point in the progression of the disorder the HoNOSCA was completed, etc.

#### Other Psychometric Characteristics

As mentioned in the Introduction section, clinicians emphasize the usefulness of HoNOSCA and its economical application (11). Also in this study HoNOSCA could be used across all disorders; clinicians saw no restrictions. Gowers et al. (3) reported a mean time to complete the scale of 8.5 min, with a range up to 18 min. Yates et al. (13) found that it took clinicians familiar with the instrument about 5 min to fill out HoNOSCA.

### Interrater Reliability

The results on intraclass correlations of HoNOSCA at the level of the individual items reported in the introduction above (3) made it clear that high interrater agreement is achievable in principle. It should be borne in mind that the frequent changes in resident and intern physicians and psychologists reduce the reliability of clinical observer ratings. Therefore, training must be provided frequently, and appropriate manuals must be made available.

## Conclusion

In sum, this study supports the validity of HoNOSCA-D. Beyond that, this study found no inconsistencies that would call into question already established qualities of HoNOSCA. Again no support was found for internal consistency of HoNOSCA-D, which is not a new finding (3, 6, 9); the HoNOSCA total score follows a different logic than the logic expressed by Cronbach’s α—namely, index construction using independent indicators. Through the low covariance between the HoNOSCA-D items, it is possible for the scale to cover a large area of application, which otherwise would only be possible through an extensive battery of tests for diagnosis.

## Ethics Statement

Ethics committee: Eidgenössische Expertenkommission für das Berufsgeheimnis in der medizinischen Forschung (Swiss federal expert committee for professional confidentiality in medical research). All entering patients were informed about the survey and could, but only if they wanted to, refuse the survey; they actively had to withdraw from the survey.

## Author Contributions

AW: theoretical backgrounds, methods, statistical analyses, reporting, and implementing these into the article. Project leader ST: statistical analyses, reporting, and implementing these into the article. RZ: critical review and correction/upgrade of the article.

## Conflict of Interest Statement

We, as authors, declare that the research was conducted in the absence of any commercial or financial relationships that could be construed as a potential conflict of interest.
